# Factors associated with oral fingolimod use over injectable disease- modifying agent use in multiple sclerosis

**DOI:** 10.1016/j.rcsop.2021.100021

**Published:** 2021-05-05

**Authors:** Jagadeswara Rao Earla, George J. Hutton, J. Douglas Thornton, Hua Chen, Michael L. Johnson, Rajender R. Aparasu

**Affiliations:** a Pharmaceutical Health Outcomes and Policy, College of Pharmacy, University of Houston, TX, USA; b Baylor College of Medicine, Houston, TX, USA

**Keywords:** Multiple sclerosis, Disease modifying agent (DMA), Fingolimod, Oral DMA, Injectable DMA, Treatment selection, MS, Multiple Sclerosis, DMA, Disease-modifying agent, FDA, Food and Drug Administration, MRI, Magnetic Resonance Imaging, ED, Emergency Department, NDC, National Drug Code, HCPCS, The Healthcare Common Procedure Coding System, ABM, The Andersen Behavioral Model, ICD-9-CM, International Classification of Diseases, Ninth Revision, Clinical Modification, CCS, Clinical Classification System, AHRQ, Agency for Healthcare Research and Quality, FIN, Fingolimod, INJ, Injectable DMAs, AOR, Adjusted Odds Ratio, SD, Standard Deviation, AV, Atrioventricular, EDSS, Expanded Disability Status Score, HMO, Health Maintenance Organization, POS, Point-of-service, PPO, Preferred Provider Organization, EPO, Exclusive Provider Organization, CDHP, Consumer Directed Health Plan, HDHP, High Deductible Health Plan, CLD, Chronic Lung Disease, DME, Durable Medical Equipment

## Abstract

**Background:**

Fingolimod is the first approved oral disease-modifying agent (DMA) in 2010 to treat Multiple Sclerosis (MS). There is limited real-world evidence regarding the determinants associated with fingolimod use in the early years.

**Objective:**

The objective of this study was to examine the factors associated with fingolimod prescribing in the initial years after the market approval.

**Methods:**

A retrospective, longitudinal study was conducted involving adults (≥18 years) with MS from the 2010–2012 IBM MarketScan. Individuals with MS were selected based on ICD-9-CM: 340 and a newly initiated DMA prescription. Based on the index/first DMA prescription, patients were classified as fingolimod or injectable users. All covariates were measured during the six months baseline period prior to the index date. Multivariable logistic regression was performed to determine the predisposing, enabling, and need factors, conceptualized as per the Andersen Behavioral Model (ABM), associated with prescribing of fingolimod versus injectable DMA for MS.

**Results:**

The study cohort consisted of 3118 MS patients receiving DMA treatment. Of which, 14.4% of patients with MS initiated treatment with fingolimod within two years after the market entry, while the remaining 85.6% initiated with injectable DMAs. Multivariable regression revealed that the likelihood of prescribing oral DMA increased by 2–3 fold during 2011 and 2012 compared to 2010. Patients with ophthalmic (adjusted odds ratio [aOR]-2.60), heart (aOR-2.21) and urinary diseases (aOR-1.37) were more likely to receive fingolimod. Patients with other neurological disorders (aOR-0.50) were less likely to receive fingolimod than those without neurological disorders. Use of symptomatic medication (for impaired walking (aOR-2.60), bladder dysfunction (aOR-1.54), antispasmodics (aOR-1.48), and neurologist consultation (aOR-1.81) were associated with higher odds of receiving fingolimod. However, patients with non-MS associated emergency visits (aOR-0.64) had lower odds of receiving fingolimod than those without emergency visits.

**Conclusions:**

During the initial years after market approval, patients with highly active MS were more likely to receive oral fingolimod than injectable DMAs. More research is needed to understand the determinants of newer oral DMAs.

## Highlights

• During 2010–2012, nearly one in seven MS patients were prescribed oral fingolimod
• Several patient and clinical factors were associated with fingolimod prescribing
• Patients with heart, eye, and urinary disease were more likely to receive fingolimod than those without those disorders
• Patients with other neurological disorders had lower odds of receiving fingolimod than those without neurological disorders
• Patients using MS symptomatic medication were more likely to receive fingolimod than their counterparts

## 1 Introduction

Fingolimod was the first oral Disease-Modifying Agent (DMA) approved by the Food and Drug Administration (FDA) in September 2010 to treat relapsing-remitting form of Multiple Sclerosis (MS). Prior to fingolimod approval, for almost two decades, only injectable DMAs – Interferon beta (1993) and glatiramer (1996) – were available to treat MS. Although, a few intravenous DMAs – mitoxantrone (2000) and natalizumab (2006) – were available to treat MS, they were not first-line agents to treat MS.^1^ Evidence indicates that fingolimod is comparable or superior to injectable DMAs in reducing relapses, delaying disability progression, and decreasing accumulation of magnetic resonance imaging (MRI) lesions. However, the side effect profile of fingolimod is extensive, and it requires more monitoring than injectable DMAs.^2^, ^3^, ^4^, ^5^, ^6^, ^7^ In addition to being effective, fingolimod's once-daily dosing offers a convenient administration schedule and facilitates better adherence than injectable DMAs.^2^^,^^3^

Fingolimod's approval provided clinicians with an additional option of DMA to treat patients with MS. After fingolimod, several other oral DMAs were approved into the market between 2012 and 2020, including teriflunomide, dimethyl fumarate, cladribine, siponimod, diroximel fumarate, ozanimod, and monomethyl fumarate.^8^ Since 2010, until today, there are no criteria or clinical recommendations regarding the selection of an appropriate DMA for patients with MS.^9^^,^^10^ It is suggested that selection of DMA should be individualized considering the patient's disease activity, comorbidities, symptoms, risk factors, values and preferences.^11^^,^^12^ In the absence of established clinical guidelines by national or international neurology societies regarding the selection of DMAs, the decision to choose oral fingolimod versus injectable DMA is complex considering their varied safety and efficacy profiles. DMA selection is generally assumed to be a collaborative decision based on both patient and provider preferences.^2^^,^^13^^,^^14^

A recent real-world study by Desai et al. evaluated factors associated with the prescription of oral DMAs versus injectable/infusion DMAs using commercial health insurance claims data from Aetna (2009 to 2014) and reported that patients' age and certain clinical factors were associated with the selection of oral DMA.^14^ However, Desai et al. assessed factors associated with prescription of any oral DMA (including newly approved teriflunomide [2012] and dimethyl fumarate [2013]) versus either first-line injectable/second line infusion DMAs. Previous evidence indicates that patient factors, primarily age and comorbidities, could play a role in the severity of MS^15^ and further affects DMA selection.^16^, ^17^, 18. However, there is limited real-world evidence regarding factors associated with the prescribing of first-line oral fingolimod versus first-line injectable DMAs, especially during the initial years after approval. Therefore, this study examined the factors associated with oral fingolimod prescribing over conventional injectable DMAs during the initial years after the approval. This retrospective study could help us understand the drivers for acceptance of first oral DMA by providers over injectables during the initial years after fingolimod approval.

## 2 Material and methods

### 2.1 Study design and data source

A retrospective longitudinal study was conducted using the IBM MarketScan Commercial Claims and Encounters data from 2010 to 2012. The 2010–2012 data set was selected to understand the drivers for initiation of the first oral DMA by providers during the initial years after fingolimod approval. The IBM MarketScan consists of more than 43.6 million commercially insured enrollees and provides a nationally representative sample of Americans with employer-provided health insurance. Beneficiaries are from large employers, health plans, government, and public organizations. It is a limited dataset that includes de-identified inpatient, outpatient, and pharmacy claims allowing for longitudinal analysis of health care utilization.^19^ This study was approved by the Institutional Review Board at the University of Houston under the ‘exempt’ category.

### 2.2 Study population

The study population included adults (≥18 years) diagnosed with MS and newly initiated oral fingolimod or conventional injectable DMAs starting September 21, 2010 (after fingolimod's FDA approval) until December 31, 2012. DMA initiation was evaluated based on the first prescription of DMA with a six months baseline period without DMA use. Patients with MS were identified using the International Classification of Diseases, Ninth Revision, Clinical Modification (ICD-9-CM) ‘340’ in diagnoses claims, and patients with DMA prescription were identified using National Drug Codes (NDC) in pharmacy claims or The Healthcare Common Procedure Coding System (HCPCS) codes in outpatient or inpatient encounter files. The NDC codes of medications were obtained from the Redbook. Based on the index/first DMA prescription, patients were classified as oral fingolimod or injectable users. Injectable DMA users consisted of patients who used interferon beta and glatiramer acetate. In this study, users of second-line infusion DMAs, or other newer oral DMAs introduced in later 2012 were excluded as their utilization was very minimal during the study period. The date of the first DMA prescription (oral fingolimod or injectable) was regarded as the index date. Patients were required to have continuous enrollment with the health insurance plan during the six months prior to the index date (baseline/lookback period). A detailed study design is presented in Appendix A.

### 2.3 Conceptual framework

This study was conceptualized based on the Andersen Behavioral Model (ABM) of health care utilization. According to the ABM, healthcare utilization is a function of characteristics that explain (i) predisposition of an individual to use (predisposing factors), (ii) enable or impede the use (enabling factors), and (iii) need (need factors) of healthcare services.^20^ Predisposing factors included age group, gender, and region. Enabling factors included employment status, type of health insurance plan, physician specialty coding flag, and prescription time period (prescription year). Physician specialty coding flag identifies patients who had highly-differentiated (≥70%) claims coded by specialty physicians. Need factors included prevalent comorbidities, Elixhauser score,^21^ MS-related symptoms/MS severity score (Appendix B),^22^ MS symptomatic medication, and health care utilization indicators. Comorbidities that are prevalent in patients with MS were collated from existing literature,^23^^,^^24^ and identified using ICD-9-CM codes from diagnosis files. Further, few additional comorbidities that were prevalent (>15%) in the study cohort were also identified.

All the selected comorbidities were identified using the clinical classification system (CCS) codes proposed by the Agency for Healthcare Research and Quality (AHRQ).^25^ Elixhauser index score is a weighted score of selected comorbidities that were identified based on diagnoses in healthcare records.^21^ It is widely used as a surrogate measure of comorbidity burden in observational healthcare research involving administrative data.^26^ MS-related symptoms were identified using ICD-9-CM and HCPCS codes from diagnoses or procedure claims. MS severity measure is a weighted score of selected MS-related symptoms or comorbidities (Appendix B).^22^ MS severity measure acts as a proxy measure of symptomatic burden or severity of MS; a higher score indicates a higher symptomatic burden.^27^ Additionally, MS symptomatic medications are drugs that are prescribed to alleviate MS-related symptoms, and the use of these medications indicates neurological impairment.^28^ Healthcare utilization measures include baseline relapse, neurologist consultation, magnetic resonance imaging (MRI) test (procedure group code: 216), and Emergency Department (ED) visit (procedure group code: 111) – MS-associated and non-MS associated. Claims-based relapse measure was operationally defined as (i) inpatient hospitalization or (ii) outpatient encounter followed by steroid prescription within 30 days of the encounter. Successive relapses within the next 30 days after the initial relapse were considered as a single relapse episode.^29^ All the covariates were measured during the six months baseline period prior to the index date.

### 2.4 Statistical analyses

Characteristics of oral fingolimod and injectable DMA users were compared and assessed using descriptive statistical tests such as chi-square test for categorical variables and *t*-test for continuous variables. Multicollinearity among the independent covariates was ruled out using the criteria of variance inflation factor (VIF) less than 10. Multivariable logistic regression was performed to determine the factors associated with the selection of fingolimod. The outcome variable was a binary indicator of oral fingolimod versus injectable DMA as the first DMA prescription; injectable DMA was considered as the reference category. As explained earlier, independent variables (predisposing, enabling, and need factors) in the multivariable logistic regression model were chosen based on the ABM. The sample size needed for the logistic model was 765 based on the independent variables selected for the study. All the statistical analyses were conducted using SAS 9.4 (SAS Institute, Cary, North Carolina) at a level of significance value of 0.050.

## 3 Results

**Fig. 1:**
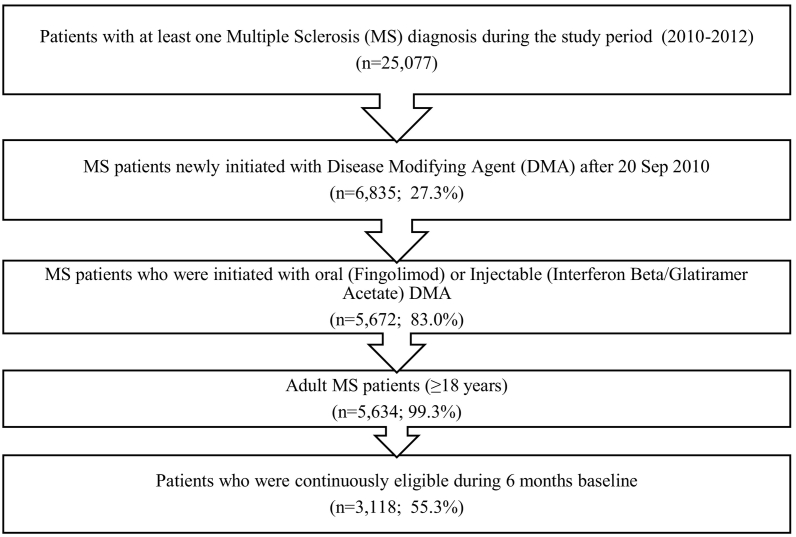
Flowchart for study cohort derivation.

The study cohort consisted of 3118 MS patients receiving DMA treatment, of which 14.4% (*n* = 450) of the individuals initiated oral fingolimod, while the remaining 85.6% (*n* = 2668) initiated injectable DMAs (See Fig. 1 for Cohort Derivation). Among injectable DMA users, 51.0% (*n* = 1360) initiated interferon-beta while 49.0% (*n* = 1308) initiated glatiramer acetate. The characteristics of the total study cohort along with the route of administration of DMA are given in Table 1**.** The cohort mainly consisted of females (77.7%), middle-aged (35–54 years; 59.9%), belonged to the South region of the US (37.6%), and were active full-time employees (80.4%) with Preferred Provider Organization (PPO) health insurance plan (60.7%). Among MS patients treated with DMAs, oral fingolimod and injectable DMA users were significantly different based on the distribution of a few predisposing (age group), enabling (employment status and prescription time period), and need factors (comorbidities, symptoms, symptomatic medication, and healthcare utilization) as shown in Table 1.

**Table 1:** Characteristics of oral fingolimod and injectable DMA users with MS: IBM MarketScan 2010–2012 (*n* = 3118).

Characteristic	Route of administration			
	Oral Fingolimod Users (N = 450; 14.4%)	Injectable DMA Users (N = 2668; 85.6%)	Total (N = 3118; 100.00%)	*p*-Value (Chi-square or t-test)
	N (%)	N (%)	N (%)	
**Predisposing factors**				
Age (in years, Mean ± SD)	45.4 ± 10.0	43.1 ± 10.9	43.4 ± 10.8	**<0.050**
*Age Group (in years)*				**<0.050**
18–34	68 (15.1%)	635 (23.8%)	703 (22.6%)	
35–44	128 (28.4%)	800 (30.0%)	928 (29.8%)	
45–54	168 (37.3%)	770 (28.9%)	938 (30.1%)	
55–64	86 (19.1%)	463 (17.4%)	549 (17.6%)	
*Gender*				0.755
Male	103 (22.9%)	593 (22.3%)	696 (22.3%)	
Female	347 (77.1%)	2075 (77.8%)	2422 (77.7%)	
*Region*				0.402
Northeast	70 (15.6%)	503 (18.9%)	573 (18.4%)	
North central	96 (21.3%)	540 (20.2%)	636 (20.4%)	
South	177 (39.3%)	995 (37.3%)	1172 (37.6%)	
West	107 (23.8%)	630 (23.6%)	737 (23.6%)	

**Enabling factors**				
*Employment Status*				**0.029**
Active full time	345 (76.7%)	2163 (81.1%)	2508 (80.4%)	
Others*	105 (23.3%)	505 (18.9%)	610 (19.6%)	
*Plan Indicator*				0.621
HMO	64 (14.2%)	394 (14.8%)	458 (14.7%)	
POS, non-capitated	59 (13.1%)	293 (11.0%)	352 (11.3%)	
PPO	267 (59.3%)	1624 (60.9%)	1891 (60.7%)	
Others (EPO, POS with capitation, CDHP, HDHP)	60 (13.3%)	357 (13.4%)	417 (13.3%)	
*Physician Specialty Coding Flag*				0.796
<70% of outpatient physician records have specialty indicated	18 (4.0%)	100 (3.8%)	118 (3.8%)	
70% or more of outpatient physician records have specialty indicated	432 (96.0%)	2568 (96.3%)	3000 (96. 2%)	
*Prescription Time Period*				
2010	25 (5.6%)	372 (13.9%)	397 (12.7%)	**<0.050**
2011	287 (63.8%)	1318 (49.4%)	1605 (51.5%)	
2012	138 (30.7%)	978 (36.7%)	1116 (35.8%)	

**Need factors**				
Mean Elixhauser Score (Mean ± SD)	1.9 ± 4.2	2.0 ± 4.3	2.0 ± 4.3	0.621
Selected AHRQ CCS comorbidities that are prevalent in MS Patients (Yes/No)				
Infections	114 (25.3%)	519 (19.5%)	633 (20.3%)	**0.004**
Cancer	78 (17.3%)	368 (13.8%)	446 (14.3%)	**0.047**
*Metabolic disorders*				
Thyroid disorders	43 (9.6%)	252 (9.5%)	295 (9.5%)	0.941
Diabetes mellitus	37 (8.2%)	227 (8.5%)	264 (8.5%)	0.840
Nutritional deficiencies	30 (6.7%)	250 (9.4%)	280 (9.0%)	0.064
Lipid disorders	62 (13.8%)	354 (13.3%)	416 (13.3%)	0.769
*Mental illness*				
Anxiety	31 (6.9%)	212 (8.0%)	243 (7.8%)	0.439
Bipolar disorders	10 (2.2%)	49 (1.8%)	59 (1. 9%)	0.579
Depression	52 (11.6%)	276 (10.3%)	328 (10.5%)	0.439
*Neurological disorders*				
Paralysis	29 (6.4%)	107 (4.0%)	136 (4.4%)	**0.020**
Epilepsy	6 (1.3%)	40 (1.5%)	46 (1.5%)	0.787
Convulsions	7 (1.6%)	64 (2.4%)	71 (2.3%)	0.267
Migraine headache	23 (5.1%)	234 (8.8%)	257 (8.2%)	**0.009**
Other headaches	45 (10.0%)	470 (17.6%)	515 (16.5%)	**<0.050**
Eye disorders	210 (46.7%)	750 (28.1%)	960 (30.8%)	**<0.050**
Ear disorders	44 (9.8%)	429 (16.1%)	473 (15.2%)	**0.001**
Other neurological disorders†	181 (40.2%)	1529 (57.3%)	1710 (54.8%)	**<0.050**
*Circulatory/vascular disorders*				
Hypertension	68 (15.1%)	426 (16.0%)	494 (15.8%)	0.646
Heart diseases	113 (25.1%)	421 (15.8%)	534 (17.1%)	**<0.050**
Cerebrovascular disease	29 (6.4%)	286 (10.7%)	315 (10.1%)	**0.005**
*Respiratory disorders*				
Chronic lung disease (CLD)	11 (2.4%)	45 (1.7%)	56 (1.8%)	0.263
*Gastrointestinal disorders*				
Liver diseases	7 (1.6%)	64 (2.4%)	71 (2.3%)	0.267
*Genitourinary disorders*				
Diseases of the urinary system	117 (26.0%)	469 (17.6%)	586 (18.8%)	**<0.050**
Complications related to pregnancy/childbirth [in females only]	12 (2.7%); [11 (3.2%); *n* = 157]	147 (5.5%) [146 (7.04%); *n* = 2265]	159 (5.1%) [157 (6.48%); *n* = 2422]	**0.011** **[0.009]**
Diseases of the skin & subcutaneous tissue	85 (18.9%)	341 (12. 8%)	426 (13.7%)	**0.001**
*Musculoskeletal disorders*				
Non-traumatic joint disorders	79 (17.6%)	463 (17.4%)	542 (17.4%)	0.917
Spondylosis, intervertebral disc disorders, other back problems	133 (29.6%)	1052 (39.4%)	1185 (38.0%)	**<0.050**
Other connective tissue diseases (including fibromyalgia)	116 (25.8%)	794 (29.7%)	910 (29.2%)	0.086
*Ill-defined conditions*				
Nausea, vomiting/abdominal pain	47 (10.4%)	295 (11.1%)	342 (11.0%)	0.701
*MS related Symptoms/Mobility Impairment*				
*MS related symptoms*	280 (62.2%)	1718 (64.4%)	1998 (64.1%)	0.375
Bladder/bowel symptoms	53 (11. 8%)	192 (7.2%)	245 (7.7%)	**0.001**
Brainstem symptoms	48 (10.7%)	436 (16.3%)	484 (15.5%)	**0.002**
Cerebellar symptoms	60 (13.3%)	373 (14.0%)	433 (13.9%)	0.713
Cerebral symptoms/cognitive impairment	13 (2.9%)	91 (3.4%)	104 (3.34%)	0.568
Difficulty walking/gait problems	44 (9.8%)	224 (8.4%)	268 (8.6%)	0.333
General symptoms	68 (15.1%)	500 (18.7%)	568 (18.2%)	0.065
Pyramidal symptoms	50 (11.1%)	229 (8.6%)	279 (9.0%)	0.082
Sensory symptoms	7 (1.6%)	45 (1.7%)	52 (1. 7%)	0.841
Speech symptoms	65 (14.4%)	827 (31.0%)	892 (28.6%)	**<0.050**
Visual symptoms	93 (20.7%)	406 (15.2%)	499 (16.0%)	**0.004**
*Mobility impairment/ Durable Medical Equipment (DME)*	26 (5.8%)	113 (4.2%)	139 (4.5%)	0.143
MS severity measure (Mean ± SD)	1.6 ± 1.9	1.7 ± 2.0	1.7 ± 2.0	0.704
*MS Symptomatic Medication*				
Analgesics	345 (76.7%)	2022 (75.8%)	2367 (75.9%)	0.687
Anticonvulsants	106 (23.6%)	535 (20.1%)	641 (20.6%)	0.089
Antidepressants	261 (58.0%)	1432 (53.7%)	1693 (54.3%)	0.088
Bladder dysfunction drugs	135 (30.0%)	423 (15.9%)	558 (17.9%)	**<0.050**
Cognition drugs	12 (2.7%)	57 (2.1%)	69 (2.2%)	0.479
Erectile dysfunction drugs	19 (4.2%)	97 (3.6%)	116 (3.7%)	0.543
Fatigue drugs	134 (29.8%)	625 (23.4%)	759 (24.3%)	**0.004**
Impaired walking drugs	102 (22.7%)	190 (7.1%)	292 (9.4%)	**<0.050**
Spasticity drugs	308 (68.4%)	1475 (55.3%)	1783 (57.2%)	**<0.050**
*Healthcare Utilization*				
Relapse	86 (19.1%)	581 (21.8%)	667 (21.4%)	0.202
Neurologist consultation	257 (57.1%)	1328 (49.8%)	1585 (50.8%)	**0.004**
Magnetic resonance imaging (MRI)	12 (2.7%)	122 (4.6%)	134 (4.3%)	0.065
*Emergency department (ED) visits*				**0.001**
No ED visit	355 (78.9%)	1962 (73.5%)	2317 (74.3%)	
MS associated	17 (3.8%)	55 (2.1%)	72 (2.3%)	
Non-MS associated	78 (17.3%)	651 (24.4%)	729 (23.4%)	

***Abbreviations:*** SD- Standard deviation, HMO -health maintenance organization, POS – point-of-service, PPO -preferred provider organization, EPO exclusive provider organization, CDHP - consumer directed health plan, HDHP - high deductible health plan

***Footnotes:***

* Other employment status include part-time/seasonal, early retiree, long term disabled etc.

† Other neurological disorders include cerebral degeneration (unspecified), Parkinson's disease, Huntington's chorea, other choreas, neuroleptic malignant syndrome, spinocerebellar disease, trigeminal nerve disorders, other retinal disorders, other demyelinating diseases of central nervous system (neuromyelitis optica, Schilder's disease, acute transverse myelitis, other demyelinating diseases of central nervous system), epilepsy and recurrent seizures, anoxic brain damage, encephalopathy, convulsions and aphasia.

Significant *p* values are bolded.

Multivariable logistic regression findings revealed that enabling (time period) and several need factors were associated with the initiation of oral fingolimod over injectable DMAs. The findings of multivariate logistic regression are shown in Table 2**.** Compared to 2010, the odds of prescribing oral DMA were 2–3 fold higher during 2011 (adjusted odds ratio [aOR]-3.34; 95% CI: 2.13–5.24) and 2012 (aOR- 2.34; 95% CI: 1.46–3.75). Patients with eye disorders (aOR- 2.63; 95% CI: 2.08–3.31), heart diseases (aOR- 2.21; 95% CI: 1.65–2.97), and urinary diseases (aOR- 1.37; 95% CI: 1.03–1.82) were more likely to receive oral fingolimod than those who did not have those disorders. Whereas, patients with other neurological disorders (aOR- 0.50; 95% CI: 0.38–0.65) and nutritional deficiencies (aOR- 0.64; 95% CI: 0.41–0.98) were less likely to receive oral fingolimod than those without those disorders/deficiencies. Further, use of symptomatic medication for impaired walking (aOR-2.60; 95% CI: 1.90–3.58), bladder dysfunction (aOR-1.54; 95% CI: 1.17–2.02), andspasticity (aOR-1.48; 95% CI: 1.15–1.91) was associated with higher odds of receiving oral fingolimod compared to those without symptomatic medication for MS. In addition, patients who had neurologist consultation (aOR-1.81; 95% CI: 1.39–2.34) had higher odds of receiving oral fingolimod than those without neurologist consultation, while patients who had non-MS associated ED visits (aOR-0.64; 95% CI: 0.46–0.88) had lower odds of receiving oral fingolimod compared to those without ED visits.

**Table 2:** Factors associated with oral fingolimod prescription in patients with MS – findings from multivariate logistic regression: IBM marketscan 2010–2012 (n = 3118).

Characteristic	Adjusted odds ratio (95% Confidence Interval)	p-value
**Predisposing factors**		
*Age Group (in years)*		
18–34	Reference	
35–44	1.15 (0.82–1.62)	0.418
45–54	1.35 (0.96–1.92)	0.088
55–64	0.92 (0.61–1.39)	0.706
*Gender*		
Female	Reference	
Male	0.98 (0.73–1.31)	0.881
*Region*		
South	Reference	
Northeast	0.74 (0.53–1.03)	0.074
North central	1.01 (0.75–1.37)	0.935
West	0.97 (0.72–1.31)	0.845

**Enabling factors**		
*Employment Status*		
Others*	Reference	
Active full time	0.90 (0.68–1.19)	0.450
*Plan Indicator*		
PPO	Reference	
HMO	0.86 (0.61–1.22)	0.401
POS, non-capitated	1.24 (0.88–1.76)	0.221
Others (EPO, POS with capitation, CDHP, HDHP)	1.02 (0.73–1.44)	0.888
*Physician Specialty Coding Flag*		
<70% of outpatient physician records have specialty indicated	Reference	
70% or more of outpatient physician records have specialty indicated	1.28 (0.71–2.33)	0.413
*Prescription Time Period*		
2010	Reference	
2011	**3.34 (2.13–5.24)**	**<0.050**
2012	**2.34 (1.46–3.75)**	**<0.050**

**Need factors**		
Mean Elixhauser Score (Mean ± SD)	1.00 (0.96–1.03)	0.948
**Selected AHRQ CCS comorbidities that are prevalent in MS Patients (Yes/No)**		
Infections	1.26 (0.96–1.65)	0.097
Cancer	1.12 (0.82–1.53)	0.474
*Metabolic disorders*		
Thyroid disorders	0.94 (0.64–1.39)	0.775
Diabetes mellitus	0.89 (0.59–1.34)	0.577
Nutritional deficiencies	**0.64 (0.41–0.98)**	**0.041**
Lipid disorders	0.99 (0.70–1.41)	0.959
*Mental illness*		
Anxiety	0.97 (0.62–1.51)	0.882
Bipolar disorders	1.54 (0.71–3.34)	0.273
Depression	0.85 (0.58–1.26)	0.423
*Neurological disorders*		
Paralysis	1.67 (0.92–3.03)	0.090
Epilepsy	1.01 (0.38–2.70)	0.987
Convulsions	0.91 (0.38–2.22)	0.842
Migraine headache	0.64 (0.39–1.05)	0.076
Other headaches	0.73 (0.50–1.06)	0.098
Eye disorders	**2.63 (2.08–3.31)**	**<0.050**
Ear disorders	**0.63 (0.44–0.91)**	**0.013**
Other neurological disorders†	**0.50 (0.38–0.65)**	**<0.050**
*Circulatory/vascular disorders*		
Hypertension	0.86 (0.63–1.19)	0.368
Heart diseases	**2.21 (1.65–2.97)**	**<0.050**
Cerebrovascular disease	**0.62 (0.39–0.99)**	**0.043**
*Respiratory disorders*		
Chronic lung disease (CLD)	0.96 (0.58–1.56)	0.860
*Gastrointestinal disorders*		
Liver diseases	0.56 (0.24–1.32)	0.183
*Genitourinary disorders*		
Diseases of the urinary system	**1.37 (1.03–1.82)**	**0.030**
Complications related to pregnancy/childbirth	0.56 (0.29–1.08)	0.083
Diseases of the skin & subcutaneous tissue	1.33 (0.97–1.81)	0.073
*Musculoskeletal disorders*		
Non-traumatic joint disorders	1.05 (0.76–1.44)	0.773
Spondylosis, intervertebral disc disorders, other back problems	0.78 (0.60–1.02)	0.067
Other connective tissue diseases (including fibromyalgia)	0.86 (0.65–1.14)	0.302
*Ill-defined Condition*s		
Nausea, vomiting/abdominal pain	0.96 (0.65–1.41)	0.833
MS severity measure (mean ± SD)	1.01 (0.93–1.10)	0.771
*MS Symptomatic Medication (Yes/No)*		
Analgesics	0.98 (0.74–1.29)	0.893
Anticonvulsants	1.18 (0.89–1.57)	0.253
Antidepressants	1.02 (0.79–1.31)	0.882
Bladder dysfunction drugs	**1.54 (1.17–2.02)**	**0.002**
Cognition drugs	0.74 (0.35–1.54)	0.416
Erectile dysfunction drugs	0.95 (0.51–1.77)	0.873
Fatigue drugs	1.12 (0.87–1.45)	0.374
Impaired walking drugs	**2.60 (1.90–3.58)**	**<0.050**
Spasticity drugs	**1.48 (1.15–1.91)**	**0.002**
*Healthcare Utilization (Yes/No)*		
Relapse	0.72 (0.52–1.00)	0.050
Neurologist consultation	**1.81 (1.39–2.34)**	**<0.050**
Magnetic resonance imaging (MRI)	0.57 (0.29–1.10)	0.094
*Emergency department (ED) visits*		
No ED visit	Reference	
MS associated	1.68 (0.88–3.23)	0.119
Non-MS associated	**0.64 (0.46–0.88)**	**0.006**

***Abbreviations:*** HMO -health maintenance organization, POS – point-of-service, PPO -preferred provider organization, EPO exclusive provider organization, CDHP - consumer directed health plan, HDHP - high deductible health plan, SD – Standard deviation

***Footnotes:*** * Other employment status include part-time/seasonal, early retiree, long term disabled etc.

† Other neurological disorders include cerebral degeneration (unspecified), Parkinson's disease, Huntington's chorea, other choreas, neuroleptic malignant syndrome, spinocerebellar disease, trigeminal nerve disorders, other retinal disorders, other demyelinating diseases of central nervous system (neuromyelitis optica, Schilder's disease, acute transverse myelitis, other demyelinating diseases of central nervous system), epilepsy and recurrent seizures, anoxic brain damage, encephalopathy, convulsions and aphasia

Significant p values are bolded

## 4 Discussion

This study examined the factors associated with the selection of the first oral DMA fingolimod over conventional injectable DMAs during the initial years after fingolimod approval (2010−2012). Approximately 15% of the patients initiated oral fingolimod during 2010–2012. This study revealed that time period (enabling factor) and several clinical (need) factors such as comorbidities, MS symptomatic medication, and healthcare utilization were associated with the selection of oral fingolimod over injectable DMAs. As expected, the likelihood of prescribing oral fingolimod increased by more than 2–3 folds after 2010. With time s, fingolimod's availability and clinicians' or patients' experience in using fingolimod might have increased, and thereby improved the chances of adopting newer oral fingolimod into clinical practice. In addition, other physician-related factors such as scientific commitment, high prescribing volume, high exposure to marketing, and communication with colleagues could have played a role in the successful adoption of fingolimod.^18^^,^^30^

Patients with heart diseases (e.g., acute coronary syndrome, heart failure, arrhythmias, conduction disorders, and valve disorders, etc.) were more than two times more likely to receive fingolimod than those without heart diseases during 2010–2012. But, based on the evolving cardiac risk profile of fingolimod over time,^**15**^ a reverse association would be expected in more recent years (post-2012) as fingolimod is contraindicated with many cardiac conditions. The initial product monograph of fingolimod, released in September 2010, included cardiac warnings such as transient bradycardia upon first administration and atrioventricular conduction (AV) block. However, based on long-term safety studies, in April 2012, the manufacturer updated several cardiac contraindications to fingolimod. Those include second-degree or higher AV block, sick sinus syndrome or sinoatrial block, and prolonged QT interval. In addition, fingolimod is not recommended for patients who were taking antiarrhythmic medication or bradycardia inducing antihypertensive medications.^6^^,^^7^^,^^16^ This is likely to reduce prescribing of fingolimod after 2012 in MS patients with cardiac conditions.

Patients with other neurological disorders (Parkinson's disease, cerebral degeneration, Huntington's chorea, neuroleptic malignant syndrome, trigeminal nerve disorders, and other demyelinating diseases) had 50% lower odds of receiving oral fingolimod. The presence of other neurological disorders might have prompted neurologists to choose much safer and established injectable DMAs than oral fingolimod. Another important finding from this study is that patients using MS symptomatic medication were more likely to receive oral fingolimod. As observed previously, the use of medication for impaired walking,^14^ bladder dysfunction, and spasticity increased the odds of receiving oral fingolimod by 1.5–2.0 times in patients with MS. Further, patients with eye diseases and urinary diseases also had higher odds of being prescribed oral fingolimod. Ophthalmic diseases, other vision symptoms, and urinary diseases could be a part of MS clinical manifestation. Research also points to the fact that newly diagnosed symptomatic MS individuals might present with vision symptoms, urinary tract infections, or bladder/bowel dysfunction requiring symptomatic treatment.^31^^,^^32^ Hence, patients with severe symptomatic burden who are at high risk of disease progression may have been more likely to receive the newer and more effective oral fingolimod instead of injectable DMAs. Current evidence informs that fingolimod is more effective than injectable DMAs, but requires closer lab monitoring^7^^,^^33^ and is suggested for patients who can be closely monitored.^34^ Therefore, prescribing DMA for MS patients is a complex decision that requires assessing the comorbidities and MS-related symptoms along with the laboratory parameters.

Consistent with previous literature,^14^ patients who had at least one neurologist consultation during baseline were nearly two times more likely to receive oral fingolimod. Patients who had non-MS-associated ED visits were 36% less likely to receive oral fingolimod. Desai et al. reported that patients who had ED visits were nearly 1.5 times more likely to receive oral DMAs.^14^ However, in Desai et al.'s study,^14^ ED visits were not classified based on MS diagnosis, which could inform the severity of MS and further treatment selection.

Overall, several factors influenced the selection of oral fingolimod over the existing injectable DMAs. In the current study, patients with other comorbidities, MS-related symptoms, and symptomatic medication suggest a more severe form of MS, and those more disabled patients were more likely to be prescribed with oral fingolimod over conventional injectable DMAs. Also, patients with cardiac diseases were more likely to be prescribed fingolimod during the early years after its approval. However, with the evolving cardiac risk profile of fingolimod in the later years, clinicians might not favor prescribing fingolimod to patients with cardiac conditions. With monitoring requirements and evolving risk profile of fingolimod, the drivers of prescribing fingolimod might have varied in recent years. Most importantly, the prescribing of oral fingolimod increased during the study period. This is expected as both clinicians' or patients' experience with fingolimod increases over time. Other market factors, including promotional activities^35^ and market access issues, might also have influenced the adoption of newer oral fingolimod into clinical practice. The early practices may not reflect the current use due to increasing evidence and experience involving fingolimod and the introduction of more oral DMAs. Therefore, more research is needed to understand the determinants of each oral DMA selection in recent times.

### 4.1 Strengths & limitations

This is the first study to assess the factors associated with oral fingolimod versus injectable DMA prescriptions during the early years after its approval. As this study used data sources that are primarily administrative in nature, there exists an issue of unmeasured confounding. Information about race/ethnicity, MS phenotype, Expanded Disability Status Score (EDSS),^36^ and laboratory findings/MRI lesions were not available. However, the primary strength of this study is that it accounted for many MS-related clinical variables such as prevalent comorbidities, MS severity measure, and MS-related symptomatic medications. This rich source of clinical variables can be considered as the proxy for EDSS (a frequently used MS severity indicator in clinical trials)^36^ in claims data. Further, physician-related variables, laboratory test information, and other market-related factors were also not available, which could have provided more understanding of factors related to fingolimod selection. Infusions were not studied as they were infrequently used as a primary treatment option to treat MS. It should also be acknowledged that, due to small sample size (<30), other relevant comorbidities/medication use that could have had an impact on treatment selection, such as autoimmune disorders, dementia/cognitive dysfunction, and diabetics with subcutaneous insulin, could not be adjusted in the model. Further, newer oral agents were not included as this study specifically aimed to assess the factors associated with the selection of the first oral DMA, fingolimod, during the initial years after its approval. Considering the above limitations and the study population, interpretation and generalization of results should be done with caution.

## 5 Conclusion

During the initial years after market approval (2010–2012), nearly one in seven MS patients initiated treatment with the first oral DMA, fingolimod. Patients' enabling and need factors were the main drivers of oral fingolimod use over injectable DMA formulation. As the time from market entry increases, the likelihood of prescribing fingolimod increased. During the early years after its approval, patients with a highly active form of MS were more likely to receive oral fingolimod than injectable DMAs. These study findings could help clinicians in treatment decision-making and recommend policy modifications to improve DMA access. However, more research is needed to understand the determinants of oral DMA formulation selection with the introduction of several oral DMAs in recent times.

## Statement of funding source and role of sponsor

This research did not receive any specific grant from funding agencies in the public, commercial, or not-for-profit sectors.

## Declaration of interest

None.

## Figures and Tables

**Figure:**
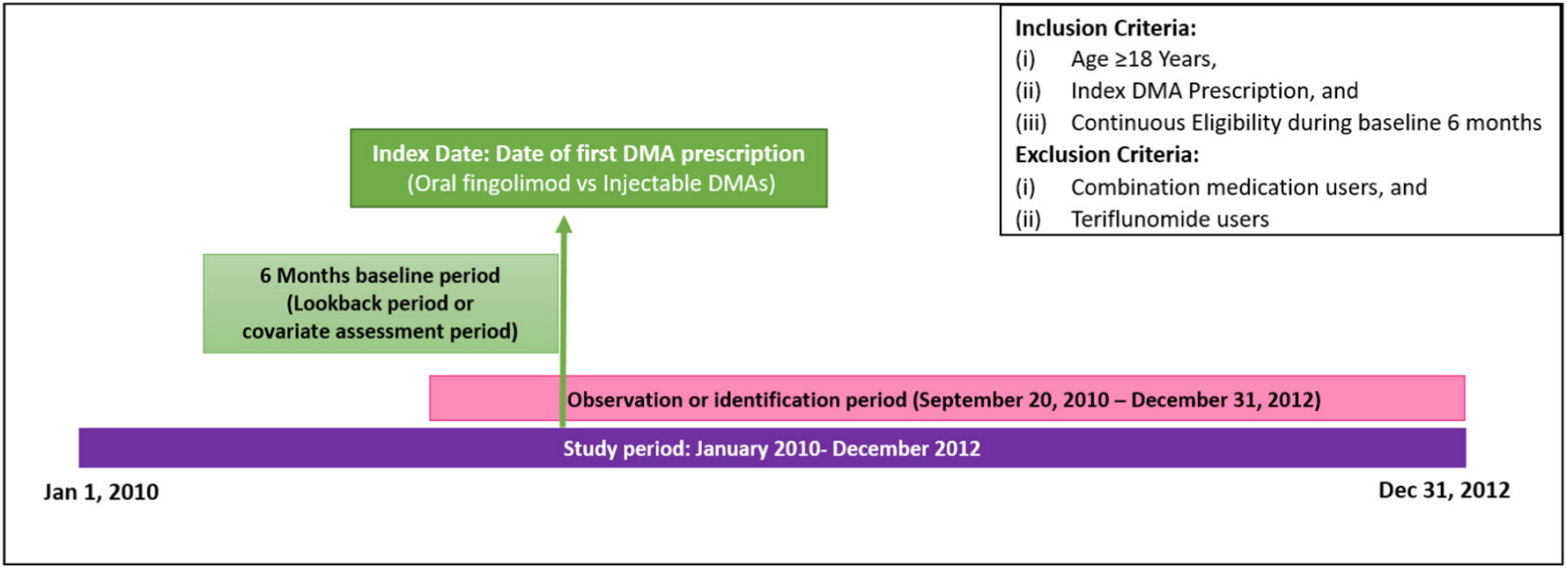


**Table:** 

S. No	MS-related symptoms	Weight
1	Bladder/bowel symptoms (incontinence/constipation) or sexual dysfunction	2
2	Brainstem symptoms (facial neuralgia, vertigo, dizziness)	1
3	Cerebellar symptoms (movement disorders, ataxia, tremor)	2
4	Cerebral symptoms/cognitive impairment (e.g. altered mental status, aphasia)	1
5	Difficulty walking/gait problems	2
6	General symptoms (fatigue)	1
7	Pyramidal symptoms (e.g. weakness, paralysis, spasticity/muscle symptoms)	2
8	Sensory symptoms (e.g. disturbances of skin sensation)	1
9	Speech symptoms	1
10	Visual symptoms (e.g. visual loss, visual disturbances)	1
11	Mobility impairment or use of Durable Medical Equipment (DME)	1
